# BTLA and PD-1 signals attenuate TCR-mediated transcriptomic changes

**DOI:** 10.1016/j.isci.2024.110253

**Published:** 2024-06-12

**Authors:** Muhammad Zainul Arifin, Judith Leitner, Donagh Egan, Petra Waidhofer-Söllner, Walter Kolch, Vadim Zhernovkov, Peter Steinberger

**Affiliations:** 1 Systems Biology Ireland, School of Medicine, University College Dublin, Belfield, Dublin 4, Ireland; 2 Center for Pathophysiology, Infectiology and Immunology, Institute of Immunology, Medical University of Vienna, Vienna, Austria; 3 Conway Institute of Biomolecular & Biomedical Research, University College Dublin, Belfield, Dublin 4, Ireland

**Keywords:** Molecular biology, Transcriptomics

## Abstract

T cell co-inhibitory immune checkpoints, such as PD-1 or BTLA, are bona fide targets in cancer therapy. We used a human T cell reporter line to measure transcriptomic changes mediated by PD-1- and BTLA-induced signaling. T cell receptor (TCR)-complex stimulation resulted in the upregulation of a large number of genes but also in repression of a similar number of genes. PD-1 and BTLA signals attenuated transcriptomic changes mediated by TCR-complex signaling: upregulated genes tended to be suppressed and the expression of a significant number of downregulated genes was higher during PD-1 or BTLA signaling. BTLA was a significantly stronger attenuator of TCR-complex-induced transcriptome changes than PD-1. A strong overlap between genes that were regulated indicated quantitative rather than qualitative differences between these receptors. In line with their function as attenuators of TCR-complex-mediated changes, we found strongly regulated genes to be prime targets of PD-1 and BTLA signaling.

## Highlights

• A human T cell reporter line co-expressing PD-1 and BTLA was generated
• RNA-seq was performed to measure transcriptome changes mediated by these receptors
• Genes induced by TCR activation were downregulated by PD-1 and BTLA signals
• TCR signaling-mediated gene suppression was attenuated by PD-1 and BTLA signals

## Graphical abstract

**Figure:**
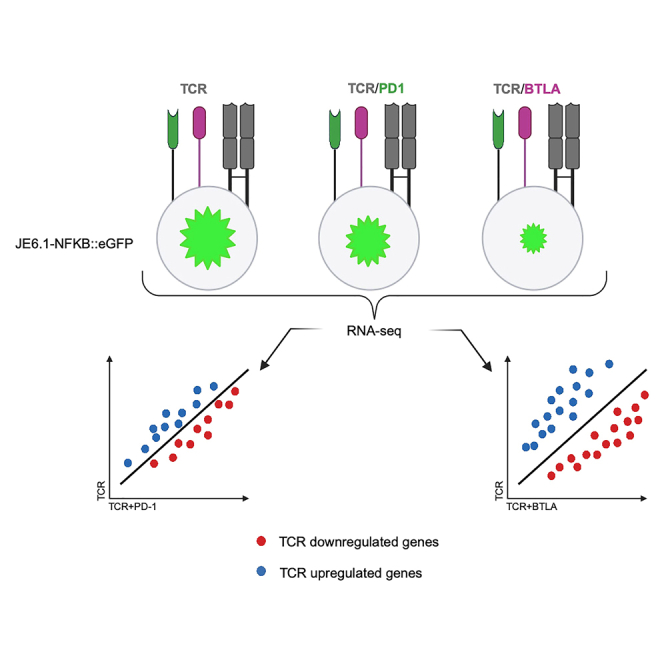

## Introduction

T cell co-inhibitory receptors, so-called immune checkpoints, play an essential role in preventing aberrant immune reactions and maintaining peripheral tolerance.^1^ However, they also limit antitumor immunity, and antibodies targeting PD-1 have revolutionized cancer therapy.^2^ Currently, Programmed cell dead protein-1 (PD-1) inhibitors are widely used to treat various malignancies such as melanoma, non-small lung cancer, and renal carcinoma. Moreover, numerous clinical trials test the utility of antibodies targeting PD-1 or its major ligand PD-L1 alone or in combination with chemotherapeutics, targeted therapies, or radiation. Nevertheless, only a subset of patients benefit from PD-1 blockade, and blocking additional immune checkpoints may increase the efficacy of immune checkpoint inhibitor (ICI) therapy. The combination of PD-1 and CTLA-4 antibodies is beneficial in some patients, and, recently, the combination of the PD-1 blocker nivolumab with the anti-LAG-3 antibody relatlimab has received Food and Drug Administration (FDA) approval for the treatment of advanced melanoma.^3^^,^^4^

B and T cell attenuator (BTLA) is a co-inhibitory receptor broadly expressed in T cells. We and others have shown that blockade of BTLA alone or in combination with PD-1 can enhance human T cell responses *in vitro*.^5^^,^^6^^,^^7^^,^^8^ However, BTLA biology is complex as its ligand Herpesvirus entry mediator (HVEM) is an activating receptor broadly expressed in T cells, where it can engage BTLA in *cis*.^9^^,^^10^^,^^11^ Consequently, the function of BTLA is controversially discussed and incompletely understood.^12^ Nevertheless, the BTLA/HVEM axis is considered an important target for cancer immunotherapy.^13^ Several BTLA antibodies have entered clinical trials where they are used alone or in combination with PD-1 antibodies.^14^^,^^15^^,^^16^^,^^17^

BTLA and PD-1 both feature a membrane-proximal immunoreceptor tyrosine-based inhibition motif (ITIM) and a membrane distal immunoreceptor tyrosine-based switch motif (ITSM). Upon tyrosine phosphorylation of these motifs, SH2 domain-containing protein-tyrosine phosphatases are recruited, which dampen signaling by the T cell receptor (TCR) and its co-activator CD28, thereby counteracting T cell activation.^2^ PD-1 mainly recruits SHP-2 (Tyrosine-protein phosphatase non-receptor type 11; PTPN11), whereas BTLA preferentially recruits SHP-1 (PTPN6).^18^^,^^19^^,^^20^ However, alternative inhibition mechanisms and even stimulatory effects have been reported for PD-1 and BTLA.^21^^,^^22^^,^^23^^,^^24^ Profiling gene expression changes mediated by co-inhibitory signals could potentially yield important insights into the function of immune checkpoints. There are several transcriptomic studies on the effects of PD-1 blockade *in vivo*, but they do not provide insight into the effects of PD-1 on TCR-induced gene expression.^25^^,^^26^^,^^27^^,^^28^^,^^29^ Studies that have investigated the direct consequences of co-inhibitory signaling on gene expression are scarce, and except for PD-1 there are no reports describing how the engagement of co-inhibitory receptors by their natural ligands affects gene expression initiated by TCR-complex signaling.^30^

We have previously used a well-controlled transcriptional reporter system engineered in the human T cell line Jurkat together with T cell stimulator cells co-expressing a membrane-bound anti-CD3 antibody fragment and different ICI ligands to study how PD-1 or BTLA impact T cell activation processes.^10^^,^^31^^,^^32^ Here, we have used our co-culture system to analyze and compare the effect of PD-1 and BTLA signaling on the transcriptional program induced by TCR signaling.

## Results and discussion

### An experimental system for dissecting the impact of PD-1 and BTLA on TCR signaling

**Figure 1:**
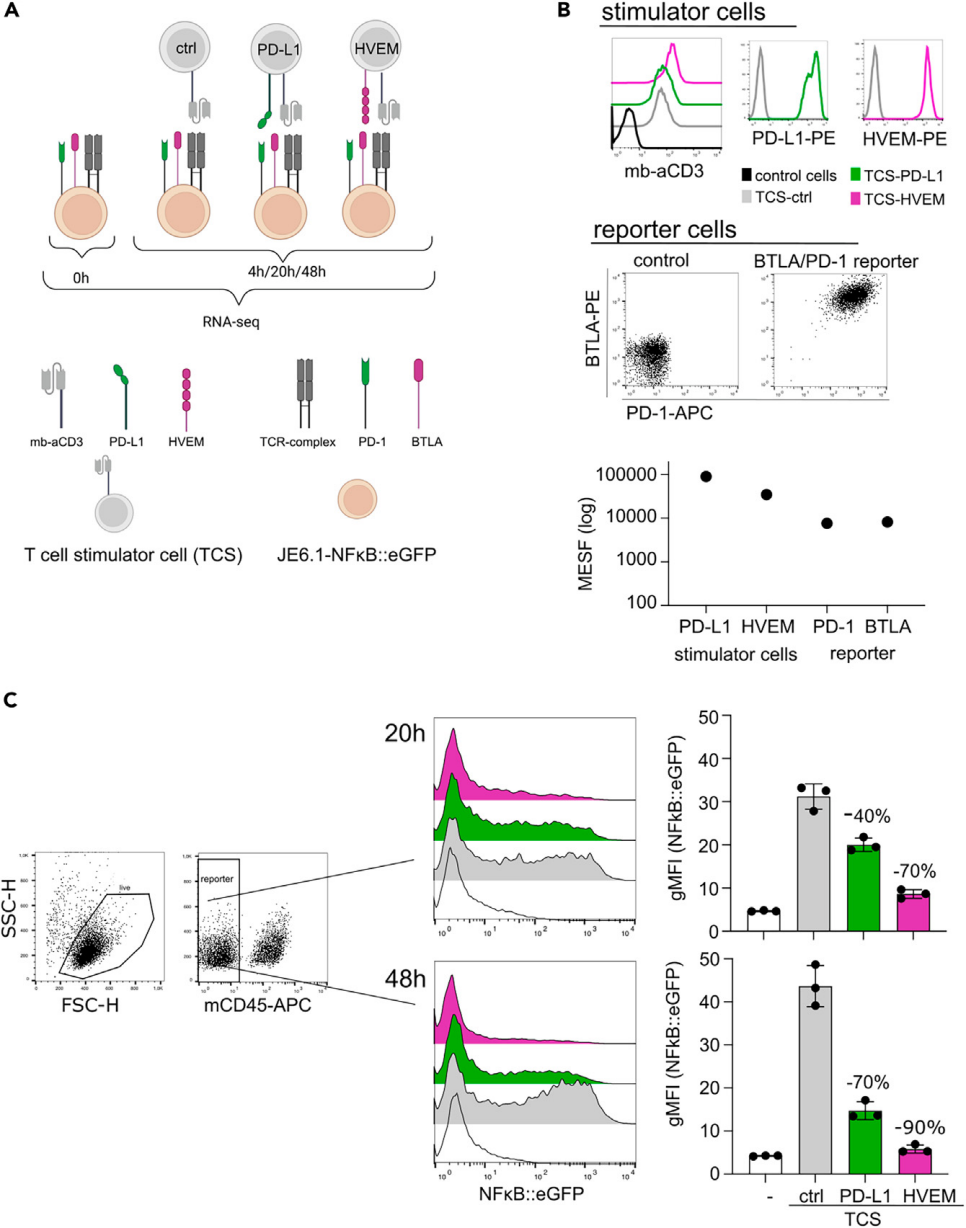
A PD-1 and BTLA co-expressing T cell reporter line for transcriptomics analyses (A) Schematic of the experimental layout. JE6.1-NF-κB::eGFP reporter cells co-expressing PD-1 and BTLA were treated as indicated. RNA was isolated from the unstimulated condition and following 4, 20, and 48 h of co-culture. (B) Flow cytometry staining of the stimulator (upper) and reporter cells (middle panel). Quantification of PD-L1 and HVEM on stimulator cells as well as PD-1 and BTLA on reporter cells using Quantum-RE-PE MESF kit is shown (lower panel). (C) Analyses of NF-κB::eGFP reporter gene induction. PD-1/BTLA-JE6.1-NF-κB::eGFP reporter T cells were co-cultured with stimulator cells (TCS) expressing PD-L1 (green bar), HVEM (magenta), or no co-stimulatory molecule (control, ctrl, gray bar) or were left untreated, respectively. The gating strategy used is shown in the left panel. TCS (expressing murine CD45) were excluded from the analysis. Reporter gene activation (eGFP) was assessed by flow cytometry following 20 and 48 h. Histograms show a representative experiment. gMFI, geometric mean fluorescence intensity. Summarized data +/− SD are shown on the right.

To assess how BTLA and PD-1 signaling impacts gene transcription induced by TCR-complex stimulation, we used a previously described Jurkat-nuclear factor κB (NF-κB)::enhanced green fluorescent protein (eGFP) reporter cell line together with T cell stimulator cells (TCS) that stimulate Jurkat T cells via the TCR-CD3 complex.^33^^,^^34^ We stably co-expressed PD-1 and BTLA in our reporter cells and generated T cell stimulator cells expressing the cognate ligands PD-L1 or HVEM (Figures 1A and 1B). Surface molecule quantitation indicated similar expression of PD-1 and BTLA on the reporter cells. On the TCS the expression of the PD-1 ligand PD-L1 was higher compared to the BTLA ligand HVEM (Figure 1B).

PD-1 and BTLA co-expressing reporter cells were left unstimulated or co-cultured with control stimulator cells or stimulator cells expressing PDL-1 or HVEM. Following 4, 20, and 48 h of co-culture, deep RNA sequencing (RNA-seq) was used to distinguish and quantify changes in gene expression in the reporter and stimulator cells.

In parallel, NF-κB::eGFP reporter gene activation was analyzed by flow cytometry. PD-1 or BTLA engagement substantially reduced reporter activation compared to control stimulation. BTLA was more potent than PD-1 (approx. 70% reduction versus 40% at 20 h), and inhibition by either receptor was more pronounced at 48 h.

### Transcriptomic changes peak at 20 h post-TCR-complex stimulation

**Figure 2:**
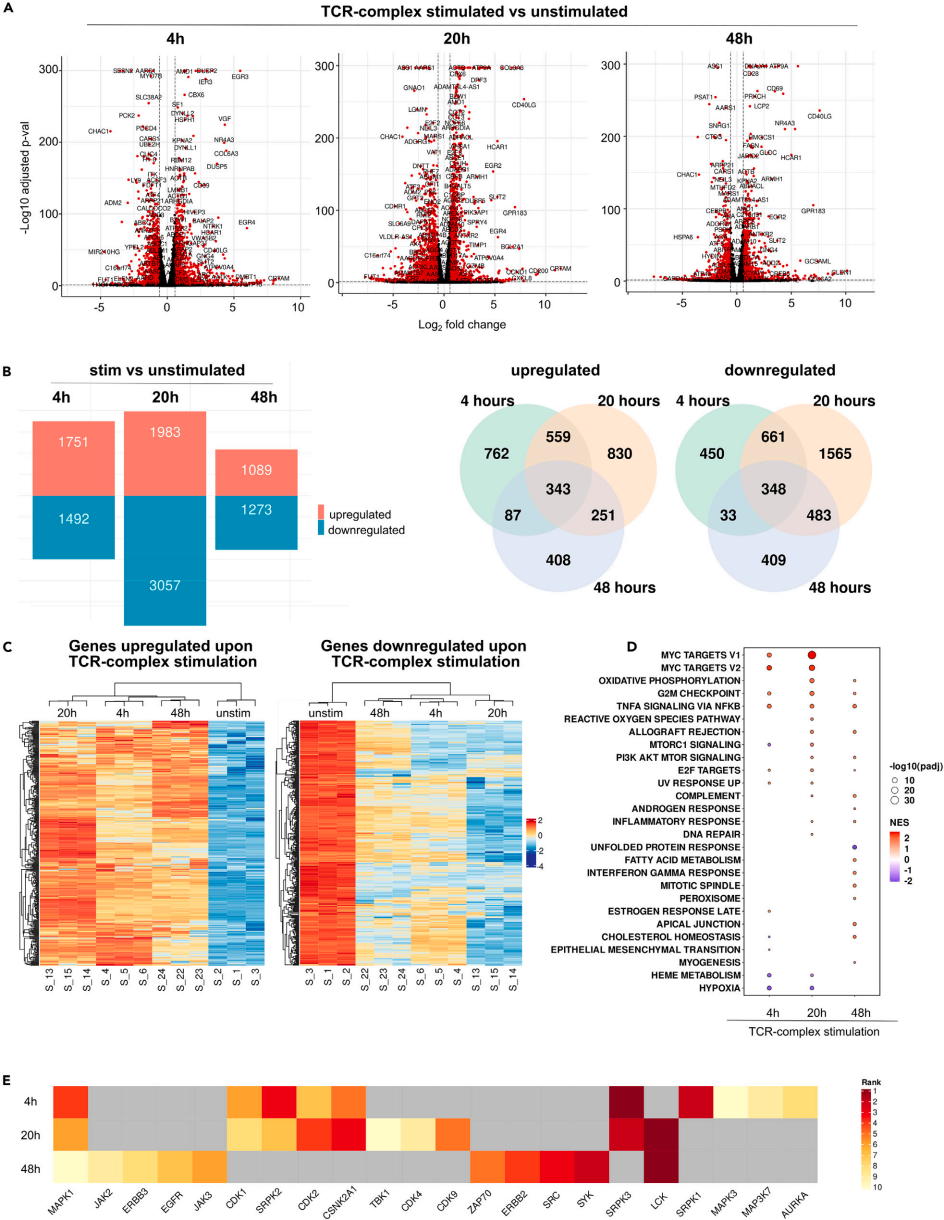
Transcriptomics analysis of TCR-complex-stimulated versus unstimulated T cell reporter cells (A) Volcano plots showing the log2-fold-change and adjusted *p* values for pairwise differential expression between stimulated T cells and unstimulated T cells across three time points (4 h, 20 h, and 48 h). Differentially expressed genes (adjusted *p* value <0.05, absolute fold-change >1.5) are highlighted in red. (B) Bar plots and Venn diagrams highlighting the number of differentially expressed genes across different time points. (C) Heatmaps of genes differentially expressed at all time points (adjusted *p* value <0.05, absolute fold-change >1.5) upon TCR stimulation. (D) Bubble plot of gene set enrichment analysis results using the Hallmark database used to identify differentially enriched pathways (adjusted *p* value <0.05) for each time point. (E) Kinase enrichment analysis of genes upregulated upon TCR-complex stimulation for all time points. The ten highest-ranked kinases were plotted in the heatmap.

Following the time course of TCR-complex stimulation, we observed an upregulation of classical T cell activation markers such as CD40LG, EGR2, and CD69 compared to unstimulated Jurkat reporter cells (Figure 2A).^35^^,^^36^^,^^37^ In total, 343 genes were significantly upregulated, and 348 genes were significantly downregulated across all time points (Figure 2B). The highest number of differentially expressed genes occurred at the 20 h time point. Interestingly, the smallest number of differentially expressed genes was found at 48 h, suggesting that the effect of TCR-complex stimulation was already diminished at that time point (Figure 2B). Hierarchical clustering of both TCR-complex-induced upregulated and downregulated genes showed that the biggest difference between unstimulated and TCR-complex-stimulated cells occurred at the 20 h time point, particularly in the number of downregulated genes (Figures 2B and 2C).

To identify biological functions associated with differentially expressed genes, we performed gene set enrichment analysis with fgsea using the Hallmark database from the Human Molecular Signature Database (MSigDB) collections (Figure 2D).^38^^,^^39^ Most significantly enriched pathways were observed at the 20 h time point. The “G2M checkpoint” and “TNFA signaling via NF-κB” were positively enriched at all time points. Identifying the “G2M checkpoint” and “E2F target functions” suggested an enrichment of the cell cycle and proliferation functions.^40^^,^^41^ The “TNFA signaling via NF-κB” pathway suggested the involvement of NF-κB in this process. Tumor necrosis factor alpha (TNF-α) can stimulate T cell expansion and functions, and the NF-κB transcription factor plays a crucial role in the TCR signaling and T cell survival and proliferation.^42^^,^^43^ Several pathways exhibited positive enrichment at specific time points. For instance, “MYC targets” and “UV response” were enriched 4 and 20 h post-stimulation, whereas “allograft rejection,” “PI3K AKT MTOR signaling,” “complement,” and “inflammatory response” were augmented at 20 and 48 h “Hypoxia” was diminished at 4 and 20 h (Figure 2D). This pathway decreases the proliferation of central memory and naive T cells after activation.^44^

Kinase enrichment analysis (KEA) identifies upstream kinases based on the overrepresentation of their putative substrates.^45^ Analyzing the list of genes upregulated upon TCR-complex stimulation, we observed changes in the activity of kinases involved in TCR signaling, such as MAPK1, CDK4, ZAP70, MAPK3, and lymphocyte-specific protein tyrosine kinase (LCK) (Figure 2E). Both LCK and ZAP70 are crucial for T cell activation. LCK phosphorylates the immunoreceptor tyrosine-based activation motifs (ITAMs) in the CD3 complex. ZAP70 then binds to these phospho-tyrosines, becomes activated, and phosphorylates adaptor proteins that mediate T cell activation.^46^

To group genes that are upregulated upon TCR-complex stimulation with similar gene expression patterns across multiple time points, we used Mfuzz clustering.^47^ Different clusters exhibited distinct signaling pathways (Figure S1A). Genes in clusters 1, 2, and 5 peaked 4 h after TCR-complex stimulation. Cluster 1 had no functional annotation, but clusters 2 and 5 contain genes with functions related to extracellular matrix (ECM) formation and organization, suggesting an enrichment of ECM-related pathways at an earlier time point. ECM-related functions facilitate cell movement, migration, and adhesion following T cell activation.^48^^,^^49^^,^^50^ Most clusters exhibited high expression levels at the 20 h time point. These clusters contained genes commonly identified in activated T cells, including NF-κB1, NF-κB2, RELA, EGR1, EGR2, DUSP2, CD69, CD27, CD40LG, TNFA, and IL2RA (Figure S1A). Gene set enrichment analysis identified pathways related to TCR signaling, cytokine production, signal transduction, and cell cycle but also to metabolic processes such RNA metabolism which facilitates the expansion and differentiation of unstimulated to activated T cells with increased proliferation and cytokine production (Figure S1B).^51^^,^^52^

### PD-1 and BTLA attenuate gene regulation mediated by TCR-complex stimulation

**Figure 3:**
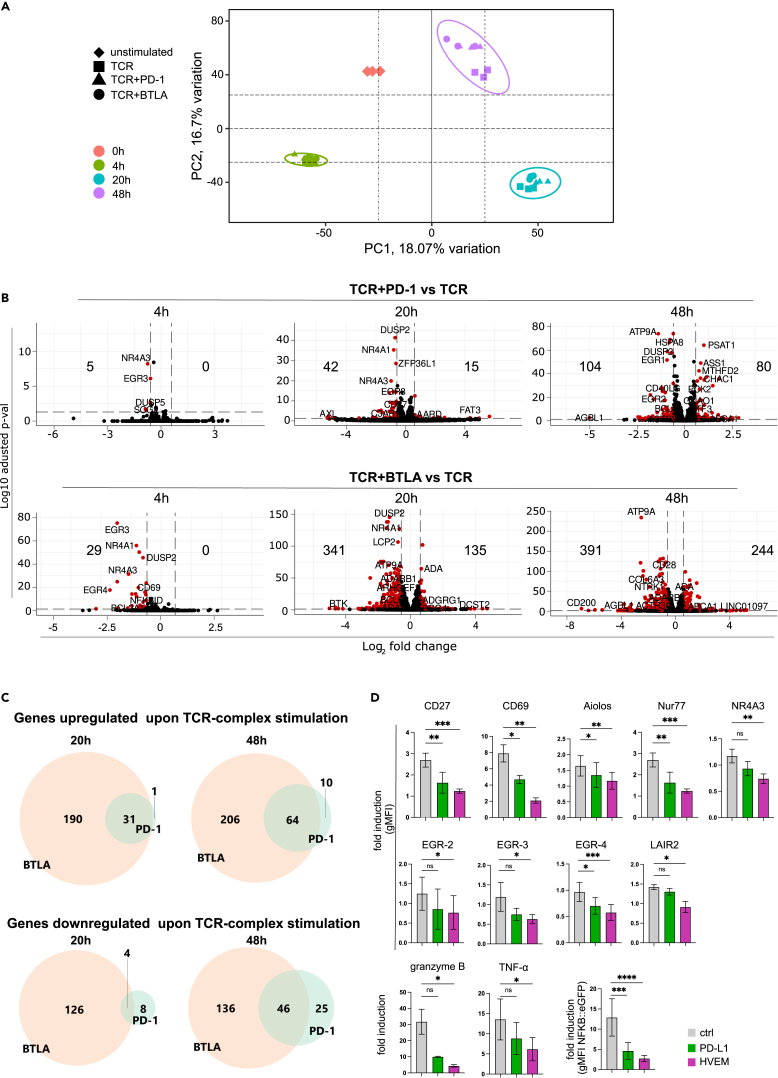
Transcriptomics changes mediated by PD-1 and BTLA signals (A) Principal-component analysis (PCA) of all samples grouped by time points and stimulation condition. (B) Volcano plot showing the log2-fold-change and adjusted *p* values for pairwise differential expression between either TCR+PD-1-stimulated or TCR+BTLA-stimulated against TCR-stimulated T cells across 3-time points (4, 20, and 48 h). Differentially expressed genes (adjusted *p* value <0.05, fold change >1.5) are highlighted in red. The number on the left side of the volcano plot indicates the amount of significantly downregulated genes whereas the number on the right side indicates the amount of significantly upregulated genes. (C) Venn diagrams of genes differentially expressed upon PD-1 inhibition and TCR stimulation intersected with genes differentially expressed upon BTLA inhibition and TCR stimulation at the 20 and 48 h time points. (D) Flow cytometry and Luminex-based analysis (granzyme B, TNF-α) showing the expression of proteins encoded by genes significantly downregulated by PD-1 and BTLA signals. Statistics were calculated using Friedman test followed by Dunn’s multiple comparison test (compared to a control group). ns, not significant; ns > 0.05, ∗*p* ≤ 0.05; ∗∗*p* ≤ 0.01; ∗∗∗*p* ≤ 0.001; ∗∗∗∗*p* ≤ 0.0001.

Principal-component analysis (PCA) of our RNA-seq data indicated that the main variation originated from the time points of stimulation (4, 20, and 48 h) rather than the type of stimulation (TCR, TCR+PD-1, or TCR+BTLA) (Figure 3A). No discernible difference was observed at the 4 h time points in the PCA plot for sample clustering. However, at 20 and 48 h the TCR-stimulated samples separated from TCR+PD-1- and TCR+BTLA-stimulated samples. To assess the impact of PD-1 and BTLA inhibition on TCR stimulation, we analyzed differential gene expression by comparing TCR-stimulated samples to TCR+PD-1-stimulated samples and TCR+BTLA-stimulated samples (Figure 3B). At 4 h, only a few differentially expressed genes were identified in PD-1- or BTLA-stimulated samples, and all were downregulated. At 20 h, PD-1 and, even more so, BTLA induced differential gene expression, and this effect was further pronounced at 48 h. This aligns with the PCA, where control-stimulated samples were most clearly separated from samples stimulated with PD-1 or BTLA ligands at 48 h. Compared to the effects of TCR-complex stimulation on gene expression, which was already very strong at 4 h and peaked at 20 h, the effects of co-inhibitory signaling are delayed, with the most profound effects occurring at 48 h.

Furthermore, there was a substantial overlap between genes regulated by PD-1 and BTLA (Figure 3C). This was most pronounced for genes upregulated upon TCR-complex stimulation and differentially expressed in PD-1- and BTLA-stimulated samples. For genes downregulated upon TCR-complex stimulation, the overlap between the differentially expressed genes upon PD-1 and BTLA treatment was less extensive but still substantial (Figure 3C). To confirm that genes regulated by PD-1 and BTLA were also differentially expressed at the protein level, we measured the expression of 11 proteins involved in T cell activation. The expression of all tested proteins was significantly reduced in our reporter cells when BTLA was engaged. Their expression was also lowered by PD-1, but the reduction did not reach statistical significance for all proteins (Figure 3D). The more substantial effect of BTLA signaling compared to PD-1 signaling was also reflected in the higher inhibition of NF-κB reporter gene expression and the much higher number of differentially expressed genes at all time points analyzed. This aligns with previous results from our laboratory that indicated a stronger inhibitory capacity of BTLA.^10^^,^^31^^,^^32^

**Figure 4:**
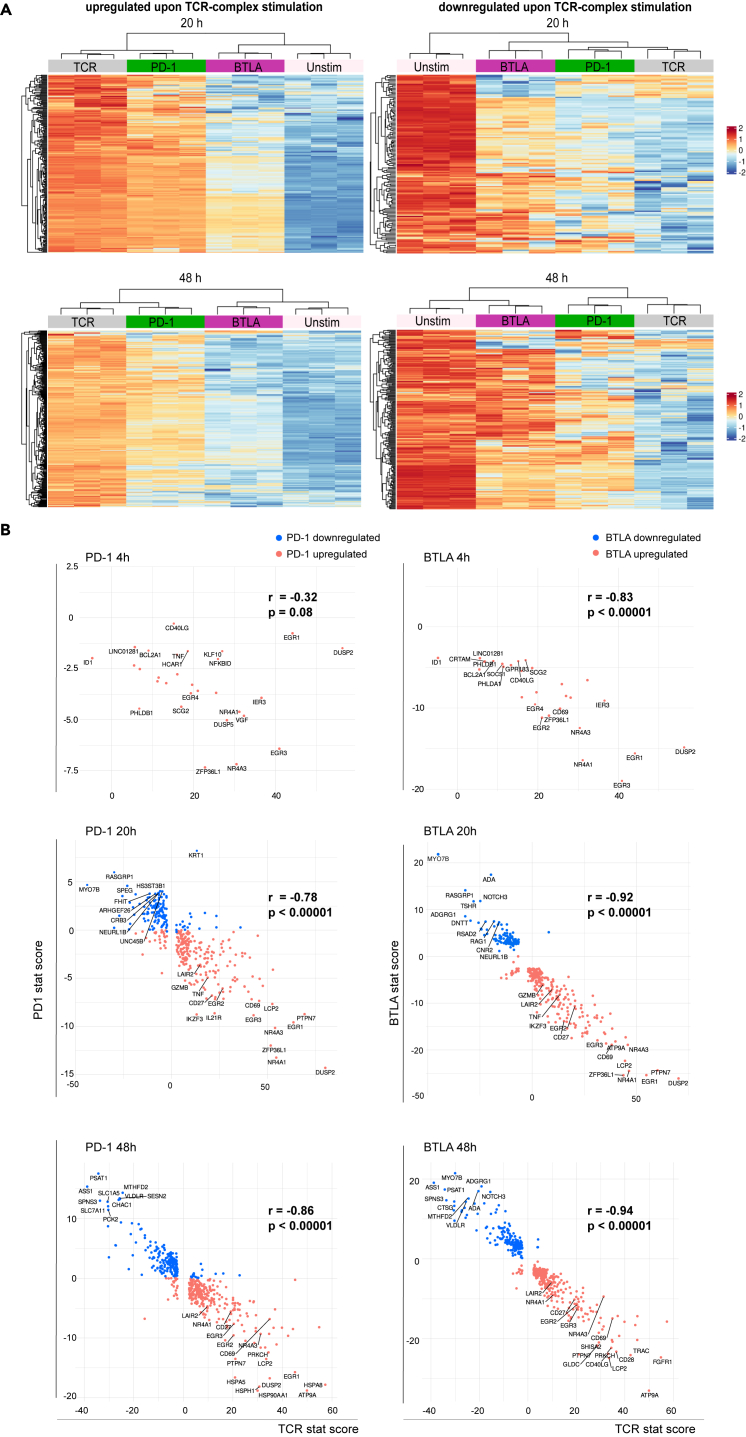
PD-1 and BTLA signals attenuate TCR-complex-mediated transcriptomic changes (A) Heatmaps of genes that are differentially expressed upon TCR-complex stimulation and significantly regulated by PD-1 or BTLA at 20 (upper panels) and 48 h (lower panels). (B) Correlation analysis of the stat scores from differential expression analysis between TCR-complex-stimulated samples against PD-1- and BTLA-regulated samples of upregulated and downregulated genes at 4, 20, and 48 h. Genes upregulated upon PD-1 or BTLA stimulation are shown in blue, whereas downregulated genes are shown in red. Spearman correlation and *p* values were indicated on the right side of each panel.

Heatmaps representing genes differentially regulated by PD-1 and BTLA showed that PD-1 and BTLA engagement downregulates genes upregulated by TCR-complex signals. Conversely, most genes suppressed by TCR-complex signals were less downregulated when PD-1 and BTLA were engaged (Figure 4A). These data indicate that PD-1 and BTLA signals attenuate transcriptomic changes mediated by TCR-complex signaling. Further correlation analysis showed that genes strongly upregulated upon TCR-complex stimulation and differentially expressed in either PD-1 or BTLA-stimulated samples were attenuated stronger by both PD-1 and BTLA. Likewise, genes strongly downregulated upon TCR-complex stimulation were preferentially higher expressed in the presence of PD-1 and BTLA signals than in control-stimulated samples (Figure 4B). These results indicate that genes strongly regulated by TCR-complex signals also tended to be subject to substantial regulation by PD-1 and BTLA signals. Even at the 4h time point, where transcriptional changes were still limited, T cell activation-related genes were already downregulated and further downregulated at the later time points. Consequently, genes prominently induced upon T cell activation, such as DUSP2, EGR1, NR4A3, and NR4A1, were also preferentially inhibited upon engagement of BTLA or PD-1 (Figure 4B). Altogether, these results suggest that both PD-1 and BTLA stimulation attenuates the effect of TCR stimulation on gene expression. The strong overlap between genes that were regulated indicated that their effect is quantitative rather than qualitative, with BTLA showing a more substantial effect than PD-1.

The most negatively enriched pathway in PD-1- and BTLA-stimulated samples was “TNFA signaling by NF-κB.” TCR signaling strongly induced this pathway, confirming that these co-inhibitory receptors counteract the transcriptomic changes mediated by TCR-complex signaling. In addition, gene set enrichment analysis showed that other pathways induced during T cell activation, such as “Myc targets,” “Interferon γ response,” “complement,” “allograft rejection,” and “inflammatory responses,” were suppressed by BTLA and PD-1 signals (Figure S2A). Interestingly, the “G2M checkpoint” and “E2F targets” were positively enriched at the 20 h time point but not at 48 h, suggesting PD-1 and BTLA repress cell proliferation-related functions at a later time point. “Apoptosis” was also negatively enriched in PD-1- and BTLA-stimulated samples (Figure S2A).

Kinases identified with KEA of genes significantly upregulated upon TCR-complex stimulation and of genes significantly downregulated by PD-1/BTLA inhibition showed that early-to-mid-TCR-signaling-related kinases, i.e., LCK, ZAP70, ITK, SYK, and MAPK1, were among the highest-ranked kinases regulated at 48 h (Figure S2B).^53^ At 48 h, a moderate correlation was observed between the kinase rank of TCR-complex stimulation and PD-1/BTLA inhibition, further substantiating the delayed inhibitory effect of PD-1 and BTLA on TCR signaling.

In total, 15 genes were significantly downregulated upon engagement of PD-1 and BTLA at the 20 and 48 h time points (Figures S3A and S3B). Importantly, all these genes were strongly induced in the reporter T cells upon activation, further corroborating that PD-1 and BTLA signals preferentially attenuate genes strongly induced by TCR-complex signaling. The identified genes and further KEA of the gene sets highlighted that PD-1 and BTLA commonly attenuate early-to-mid-TCR-signaling-related genes and kinases (Figure S3C).

When analyzing the relationship between TCR signal strength required for the induction of a gene and its sensitivity to PD-1-mediated inhibition, Shimizu et al.^30^ observed that genes induced by weak TCR signaling were resistant to PD-1 inhibition. In contrast, genes that required strong TCR signals were strongly inhibited. The authors proposed that effector molecules, such as cytokines requiring strong TCR signals, are more prone to PD-1 inhibition than genes mediating T cell differentiation and proliferation, such as transcription factors. We have not varied the TCR signaling strength in our study but observed that PD-1 and BTLA signaling strongly inhibited the expression of several transcription factors such as EGR1, EGR2, EGR3, NR4A1, and NR4A3 (Figure S4A). For instance, in our dataset, EGR1, described as refractory to PD-1 inhibition in the Shimizu study, was significantly downregulated by PD-1 and BTLA (Figure S4A).

PD-1 is upregulated upon T cell exhaustion, and PD-1 signaling significantly contributes to the exhausted state of T cells chronically exposed to antigens. Quigley et al. reported that PD-1 upregulates a program of genes in exhausted CD8 T cells in humans and mice.^54^ Among these genes was the basic leucine transcription factor ATF-like (BATF). The authors found this gene was also upregulated in PD-1-stimulated Jurkat T cells and proposed that PD-1 inhibits T cell function by upregulating BATF. Our study did not observe an active upregulation of genes upon PD-1 signaling, and PD-1 signaling failed to increase BATF expression in our Jurkat reporter T cell line (Figure S4B).

We also used cDNA obtained at 4, 20, and 48 h in qPCR experiments to validate the RNA-seq data for genes that were prominently regulated by BTLA and PD-1 signals. Our results showed that their expression peaked at 20 h corroborating that TCR/CD3-mediated changes of gene expression were most pronounced at this time point. Moreover, we observed that BTLA and PD-1 reduced the expression of these genes at each time point indicating that for strongly regulated genes the effects of inhibitory signals could already be detected at early time points (Figure S4C).

Apart from the inhibitory ITIM and ITSM motifs, the cytoplasmic domain of BTLA contains a membrane-proximal Grb-2 binding motif, which has been implicated in transmitting positive signals.^23^ Engagement of this motif could further upregulate TCR-induced genes or induce a distinct expression signature. Genome-wide transcriptional analysis revealed a co-stimulatory gene expression signature of murine CD4 T cells cultured with CD3 and BTLA antibodies immobilized on beads.^55^ By contrast, our study did not detect genes that were actively upregulated upon BTLA engagement, indicating that BTLA did not emanate activating signals strong enough to induce a distinct transcriptional program in our model.

Other immune checkpoints, like CTLA-4, TIGIT, and LAG3, also attenuate T cell activation and repress TCR signaling.^56^^,^^57^^,^^58^ CTLA-4 and TIGIT both act as a competitor to co-stimulatory receptors: CTLA-4 to CD80/CD86 that normally interact with CD28 and TIGIT to CD155/CD112 that normally interact with CD226 for T cell activation.^59^ Similar to PD-1 and BTLA, there was proof that CTLA-4 and TIGIT engagement could recruit SHP-2 even though further downstream implication is still unclear.^60^ Other immune checkpoint like LAG3 could be directly associated with TCR-CD3 complex which resulted in the dissociation of LCK from CD4 or CD8.^58^ While different immune checkpoints can have a different mechanism of actions, the following downstream events resulted in the attenuation/inhibition of T cell activation similar to what we observe on PD-1 and BTLA.

Altogether, our results do not indicate active gene regulation by PD-1 or BTLA signals. Instead, we observed an attenuation of TCR-complex-mediated transcriptional reprogramming of Jurkat T cells, which was more pronounced when BTLA was engaged although both receptors were expressed at similar levels on the reporter cells. Our results are consistent with a recruitment of SHP-1 and SHP-2, which counteract the activity of protein kinases induced by TCR-complex signaling. Celis-Gutierrez et al. have used a mass spectrometry (MS)-based approach to analyze the interaction of SHP-1 and SHP-2 with PD-1 and BTLA in primary murine CD4 T cells treated with pervanadate. They show that, while both receptors can recruit SHP-2 with similar capacity, BTLA was a better recruiter of SHP-1 and therefore afforded a better overall recruitment of inhibitory phosphatases.^61^ In accordance with these data, we observed quantitative rather than qualitative differences between these immune checkpoints, and our results show that BTLA is inducing a stronger attenuation of TCR-mediated transcriptomic changes than PD-1.

### Limitations of the study

A potential limitation of our study is that it relies on a human T cell line. We have chosen this reductionist model since it allows us to analyze the regulation of gene expression by BTLA and PD-1 in a well-controlled setting where both receptors are homogeneously expressed and exert strong inhibitory activity on T cell activation processes. We have also performed preliminary experiments where we used our T cell stimulator platform to stimulate primary human T cells in the presence of PD-1 and BTLA ligands. Engagement of both receptors inhibited T cells as shown by CD25 upregulation, and there was a tendency for stronger inhibition via BTLA (Figure S4D). We have recently shown that primary human T cells overexpressing PD-1 and BTLA were strongly inhibited in the presence of the ligands to these immune checkpoints.^62^ Human T cells engineered to co-express matched levels of PD-1 and BTLA might be useful to study and compare transcriptomic regulation mediated by PD-1 and BTLA signals in primary CD4 and CD8 T cells.

## STAR★Methods

### Key resources table

**Table:** 

REAGENT or RESOURCE	SOURCE	IDENTIFIER
**Antibodies**		

CD4 #OKT4	Biolegend	Cat#317434; RRID: AB_2562134
CD8 #HIT8a	Biolegend	Cat#300922; RRID: AB_1575072
CD25 #M-A251	Biolegend	Cat#985802
mCD45.2-APC #104	Biolegend	Cat#109814; RRID: AB_389211
DyLight-649-labeled goat-anti-mouse IgG (H+L)	Jackson ImmunoResearch	Cat#115-475-003; RRID: AB_2338786
Aiolos /IKZF3 #14C4C97	Biolegend	Cat# 371006; RRID: AB_2616804
CD14 #63D3	Biolegend	367104; RRID: AB_2565888
CD27 #M-T271	Biolegend	356406; RRID: AB_2561825
CD69 #FN50	Biolegend	310906; RRID: AB_314841
PD-L1 #29E.2A3	Biolegend	329706; RRID: AB_940368
HVEM #122	Biolegend	318806; RRID: AB_2203703
BTLA #MIH-26	Biolegend	344506; RRID: AB_2065761
PD-1 # EH12.2H7	Biolegend	329906; RRID: AB_940483
Nur77 #12.14	ebioscience	12-5965-82; RRID: AB_1257209
NR4A3 #H7833	Biotechne	PP-H7833-00
LAIR2 #319701	Biotechne	MA5-24041
EGR-2# JG78-39	Invitrogen	MA5-34770; RRID: AB_2848678
EGR-3	Bioss	BS6448R
EGR-4	Invitrogen	PA5-116975; RRID: AB_2901605
goat-anti-mouse-IgGFcγ	Jackson ImmunoResearch	Cat#115-110-071; RRID: AB_2338626
goat-anti-rabbit- IgG (H+L)	Jackson ImmunoResearch	Cat#111-116-144; RRID: AB_2337985

**Critical commercial assays**		

RNeasy	Qiagen	Cat#: 74104
Miliplex Human CD8^+^ T-Cell Magnetic Bead Panel	Merck KGaA, Darmstadt, Germany	Cat#: HCD8MAG-15K
Quantum MESF Kit, Lot 15639	Bangs Laboratories	Cat#: 827

**Deposited data**		

Gene Expression Omnibus genomics data public repository with the Accession Number GSE239586.		Accession Number GSE239586

**Experimental models: Cell lines**		

Jurkat cell line (JE6.1)	in house stock	
BW5147	in house stock	
Jurkat-NFKB:eGFP monoreporter	Jutz et al.^31^	
T cell stimulator cells	Leitner et al.^34^	

**Oligonucleotides**		

GAPDH: GAPDH-L: CGACCACTTTGTCAAGCTCA GAPDH-R: AGGGGAGATTCAGTGTGGTG	This paper	N/A
CD40L hCD40L-L: AGCCAGTTTGAAGGCTTTGT hCD40L-R: TTCAGCCCACTGTAACACAGAT	This paper	N/A
EGR1 hEGR1-L: AGCAGCACCTTCAACCCTC hEGR1-R: CCAGCACCTTCTCGTTGTTC	This paper	N/A
EGR2 hEGR2-L: TCGGTGACCATCTTTCCCAA hEGR2-R: CCAGCACCTTCTCGTTGTTC	This paper	N/A
CD69 hCD69-L: AGTCCCCATTTCTCAACACG hCD69-R: GAGAATGTGTATTGGCCTGGA	This paper	N/A
NR4A1 hNR4A1-L: GCCTGGCGTACAGGTCTAAG hNR4A1-R: CAGTTTGCCCAACAGACGTG	This paper	N/A

**Recombinant DNA**		

pCJK2 retroviral expression vector	131-141. https://doi.org/10.1016/j.jim.2010.09.020.	N/A

**Software and algorithms**		

FlowJo software version 10.4.1	Tree Star	
Bangs Laboratories/ QuickCal v3.0	Bangs Laboratories	
Graphpad Prism software version 9	GraphPad Software, Inc	
Illumina platform NovaSeq 6000 S4		
STAR aligner version 2.4.2a	Dobin et al.^63^	
htseq-count script in the HTSeq package v0.6.1	Anders^64^	

**Chemicals, peptides, and recombinant proteins**		

iQSYBR Green Supermix	BioRad	
RevertAid H Minus RT	Thermo Scientifc	EP0451

### Resource availability

#### Lead contact

Further information and request for resources, reagents and source data should be directed to and will be fulfilled by the lead contact Peter Steinberger (peter.steinberger@meduniwien.ac.at).

#### Materials availability

Plasmids used for generation of the reporter lines are available from Addgene or by the lead contact upon request. All other Plasmids used in this study are provided by the lead contact upon request.

There are restrictions to the availability of the Jurkat-reporter and Stimulator cell lines due to a material transfer agreement (MTA).

#### Data and code availability

• Any additional information required to reanalyze the data reported in this study is available from lead contact upon request.
• No original code was generated in this study.
• RNA-Seq data have been deposited in the Gene Expression Omnibus genomics data public repository with the Accession Number GSE239586. The code to perform the analysis and generate the figures is publicly available at https://github.com/ZainulArifin1/Jurkat_Transcriptomics_Analysis.

### Experimental model and study participant details

#### Human peripheral blood samples

The study with peripheral blood mononuclear cells (PBMCs) was approved by the ethics committee of the Medical University of Vienna (ECS 1083/2016). Informed written consent was obtained from all individuals. Heparinized whole blood (leucocytes reduction chambers) of healthy donors was purchased from the general hospital in Vienna, Austria (AKH; blood transfusion department). PBMCs were isolated by standard density-gradient centrifugation using Lymphoprep solution (Technoclone, Austria).

#### Cell lines

Jurkat E6.1 and the mouse thymoma Bw5417 cell line (called BW here) were derived from in house stocks. The monoreporter cell line is a Jurkat E6.1 cell harboring an NFkB::eGFP reporter construct.^31^ T cell stimulator cells (called TCS here) are BW cells that can stimulate the TCR-complex of the Jurkat reporters via membrane-bound CD3 single-chain antibody fragments (clone OKT3) fused to a CD14 stem.^34^

### Method details

#### Cell culture, antibodies and flow cytometry

Jurkat E6.1 and the mouse thymoma Bw5417 cell line were cultured in RPMI1640 containing 10% FCS, 100 μg/mL streptomycin and 100 U/mL penicillin at 37°C and 5 % CO_2_. as described (33). Antibodies used in this study are summarised in the key resources table. BD Cytofix/Cytoperm (BD Biosciences, Franklin Lakes, NJ) was used following the manufacturers’ instructions for intracellular staining.

To quantify surface expression of PD-L1 and HVEM on the stimulator cells and PD-1 and BTLA on the reporter cells Quantum-RE-PE MESF kit (Bands Laboratories Inc.) was used according to the manufacturers’ instructions.

Flow cytometry analysis was performed using FACSCalibur™(BD Bioscience) or CytoflexS (Beckmann Coulter, CA, USA) flow cytometers. FlowJo software (v10.4.1. Tree Star, Ashland, OR) was used for flow cytometry data analysis. FACS data is shown as gMFI (geometric mean fluorescence intensity). In Figure 3D, gMFI was normalised to gMFI of unstimulated condition and flow cytometry data was expressed as fold induction.

#### Generation of T-cell stimulator and Jurkat reporter cell lines

Sequences encoding BTLA (Uniprot Q7Z6A9) and PD-1 (Uniprot Q15116) were cloned into the retroviral expression vector pCJK2 and co-expressed in a previously described Jurkat E6-1-NFkB::eGFP reporter cell line.^31^ pCJK2 expression vectors encoding PD-L1 (Uniprot Q9NZQ7) or HVEM (Uniprot Q92956) were expressed in T-cell stimulator cells (TCS) as described previously.^33^

#### Reporter assay

For a standard reporter assay Jurkat-NFkB::eGFP reporter cells co-expressing PD-1 and BTLA (5x10^4^ in 50 μl) were co-cultured with TCS-control, TCS-PD-L1 or TCS-HVEM (2x10^4^ in 50 μl) in a 96-well round bottom and incubated for 24h (reporter: TCS ratio 2.5:1).

Reporter gene induction is shown as gMFI (geometric mean fluorescence intensity). For some experiments, reporter gene induction in response to stimulation was normalised to unstimulated reporter cells as indicated and expressed as fold induction.

#### RNA isolation and real-time PCR

For RNA isolation Jurkat-NFkB::eGFP reporter cells co-expressing PD-1 and BTLA were left unstimulated or were incubated with TCS-control, TCS-HVEM or TCS-PD-L1 cells at a ratio 2.5:1 (0.2x10^6^ reporter + 0.08x10^6^ TCS in a 48-well). RNA was isolated from unstimulated cells (0 h) and at 4, 20 and 48 hours after stimulation using RNeasy according to the manufacturers’ instructions (Qiagen, Germany). In parallel, NFkB::eGFP reporter activity was analysed by flow cytometry after 20 and 48 hours as described previously.^31^

In addition cDNA was prepared of each time point. qPCR primers were designed with Primer Blast (see key resources table). For Real-time PCR iQ SYBR-Green SuperMix (Biorad) was used. RT-PCR analyses was performed on a CFX Opus 96 Real -Time PCR System from BioRad. For standardization of gene expression GAPDH was used. Target gene expression levels were normalized to the house-keeping gene and expressed as fold induction to unstimulated condition using the 2^-ΔΔCT^ method.

#### Luminex

Supernatants of reporter assays were harvested following 20 h of co-culture. Granzyme B and TNF-α were measured with the Luminex 100 system (Luminex Inc., Texas, USA) according to the manufacturers’ instructions.

#### RNA-seq library preparation

The purification of messenger RNA (mRNA) from total RNA was performed using poly-T oligo-attached magnetic beads. Following fragmentation, the first strand cDNA was synthesised using random hexamer primers and subsequently subjected to second-strand cDNA synthesis, followed by end repair, A-tailing, adapter ligation, size selection, amplification, and purification. Library quality was assessed using Qubit, quantitated by real-time PCR, and assessed for size distribution by bioanalyzer. The quantified libraries were pooled and sequenced on an Illumina platform NovaSeq 6000 S4 with paired-end 2 ×150 bp runs at a depth of 50 million paired reads per sample.

#### Reference genomes for read alignment

Two reference genomes were used: the Human Reference Genome (hg38), hereafter referred to as HRG; and the mouse reference genome (mm39). To distinguish the mouse chromosomes, they were renamed as “m.chr”. The human and mouse fasta files were concatenated and indexed using the appropriate tool provided by each aligner.

#### RNA-seq processing

RNA-seq processing involved the following steps (i) raw sequence reads were quality checked using fastqc v0.12.1 (http://www.bioinformatics.babraham.ac.uk/projects/fastqc/) and low-quality reads were trimmed using BBDuk (http://jgi.doe.gov/data-and-tools/bb-tools/); (ii) alignment to the Human-Mouse reference genomes was performed using STAR v2.4.2a;^63^ (iii) counting of reads aligned over exonic features for gene expression quantification was performed using the htseq-count script in the HTSeq package v0.6.1 in the ‘Union’ overlap resolution mode.^64^ The Gene Transfer Format (GTF) file used for counting was a merged Homo sapiens and Mus musculus GTF file, both obtained from GENCODE (https://www.gencodegenes.org/pages/faq.html), and modified to ensure chromosomal compatibility with the Human-Mouse reference genome; (iv) counts were assembled using featureCounts.^65^

#### Identification of differentially expressed genes

All downstream computational analyses were performed using R statistical software version 4.1.2.^66^ To identify differentially expressed genes (DEGs) upon TCR-complex stimulation, differential expression analysis was performed between TCR-complex stimulated samples (4, 20, and 48 hours) against unstimulated samples using DESeq2 v1.34.0.^67^ To find out the effect of PD-1 and BTLA inhibition, differential expression analysis was conducted with the following experimental design “TCR+PD-1 vs TCR” and “TCR+BTLA vs TCR” for all time points. Differentially expressed genes were denoted by an absolute fold-change of > 1.5 and adjusted p-value < 0.05.

#### Gene set enrichment analysis (GSEA)

The result of differential expression analysis, particularly the stat value for each gene, was used for gene set enrichment analysis using fgsea v1.25.1.^68^ The default fgsea parameters were used except the minSize parameter, which was set to 15. The hallmark gene set from the Human Molecular Signature Database (MSigDB) was used as a ref.^38^^,^^39^

#### Gene expression clustering analysis

MFuzz v2.54 was employed to cluster genes with similar expression patterns across time points for all stimulation conditions.^47^ For TCR-complex stimulated samples, DEGs (upregulated or downregulated) were obtained for all time points. The normalised counts for each gene were aggregated by the mean based on the time point. Next, the aggregated data was zero-mean normalised, followed by the optimum number of clusters determination. Finally, Mfuzz clustering and visualisation were performed. For PD-1 and BTLA-inhibited samples, following mean aggregation, the matrix from either PD-1 or BTLA-inhibited was subtracted with the aggregated TCR matrix to eliminate the effect of TCR-complex stimulation. The processing steps were the same as TCR-complex stimulated samples.

#### Gene sets functional enrichment

Following MFuzz clustering, gene sets from each MFuzz cluster for all conditions were subjected to functional enrichment using gProfiler2 v0.2.1.^69^ Reactome and KEGG pathway annotations were used for this analysis.^70^^,^^71^

#### Kinase enrichment analysis

Genes upregulated upon TCR-complex stimulation and concomitantly differentially expressed in either PD-1 or BTLA stimulation were subjected to kinase enrichment analysis with KEA3 (https://maayanlab.cloud/kea3/).^45^ The top 300 most significant genes were collected based on the adjusted p-value for differentially expressed at each time point at the aforementioned conditions. Then, genes were subjected to the over-representation analysis for kinase enrichment. The top high-confidence 10 kinases based on the mean-rank scoring were selected and visualised.

### Quantification and statistical analysis

Statistical analyses were performed using GraphPad Prism (Version 9, GraphPad Software, Inc., La Jolla, CA, USA). In Figure 3 statistics were calculated using Friedman test followed by Dunn’s multiple comparison test (compared to a control group) and mean +/- SD are shown. In Figure S4D One-way Anova followed by Dunnett’s multiple comparison (compared to control stimulation) was used. ns, not significant; ns > 0.05, ∗p ≤ 0.05; ∗∗p ≤ 0.01; ∗∗∗p ≤ 0.001; ∗∗∗∗p ≤ 0.0001.
