# BTLA and PD-1 signals attenuate TCR-mediated transcriptomic changes

**DOI:** 10.1016/j.isci.2025.111773

**Published:** 2025-01-11

**Authors:** Muhammad Zainul Arifin, Judith Leitner, Donagh Egan, Petra Waidhofer-Söllner, Walter Kolch, Vadim Zhernovkov, Peter Steinberger

## Main text

(iScience *27*, 110253; July 19, 2024)

In the originally published version of this article, the inset legend for the graphs in Figure 4B was unintentionally flipped during figure preparation. The correct color coding for PD-1 or BTLA downregulated (red) and upregulated (blue) has been corrected online. This change does not affect the scientific conclusions, and the authors have approved the correction. The authors apologize for any confusion caused by this error.

**Figure:**
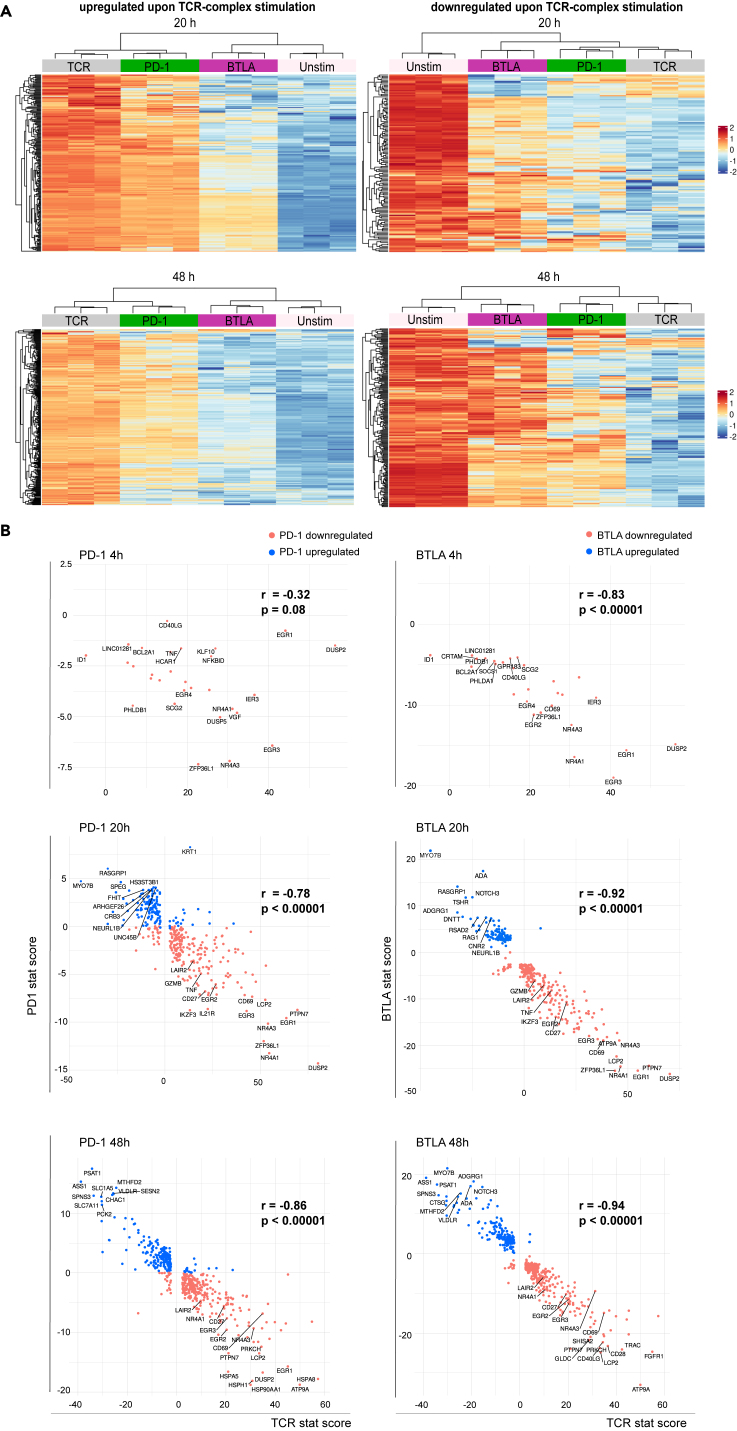
Figure 4. PD-1 and BTLA signals attenuate TCR-complex-mediated transcriptomic changes (corrected)

**Figure:**
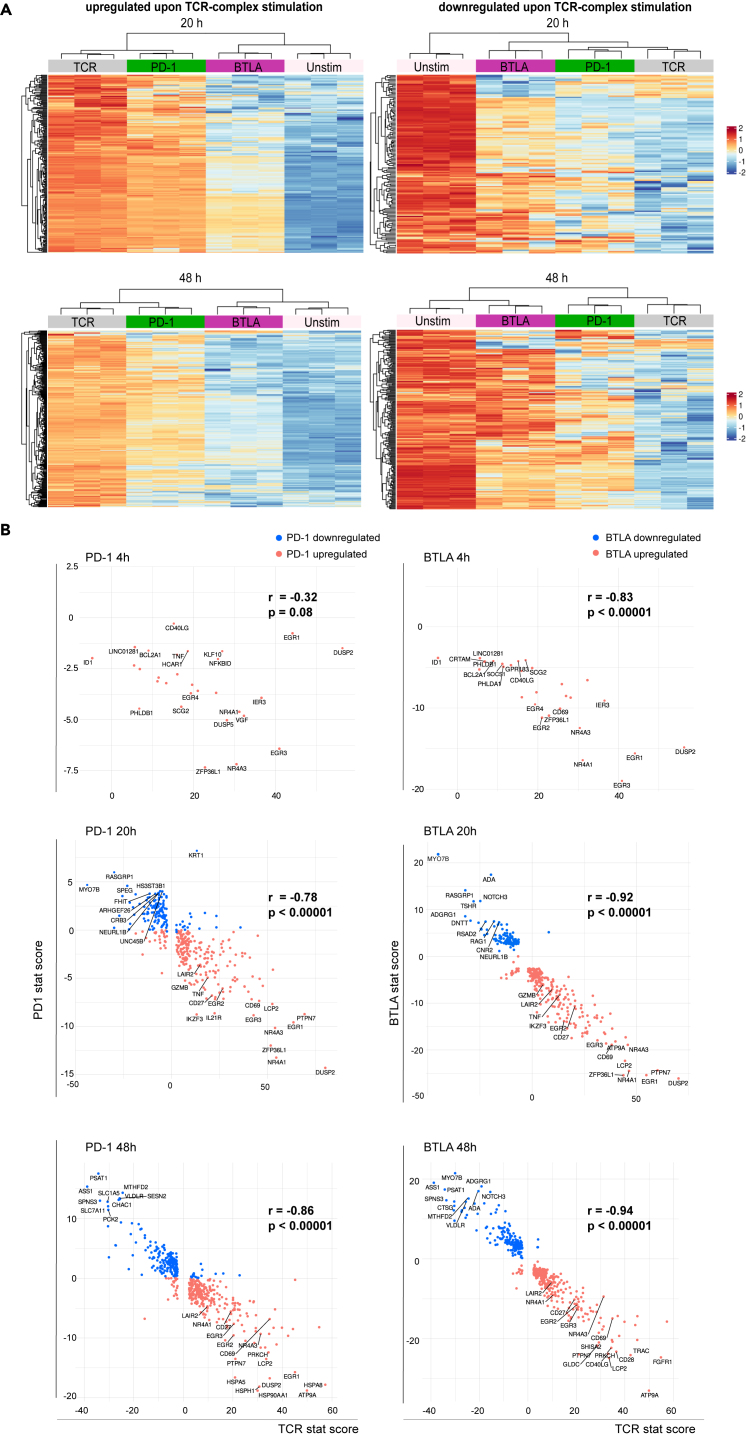
Figure 4. PD-1 and BTLA signals attenuate TCR-complex-mediated transcriptomic changes (original)

