# Supplementary material for: BTLA and PD-1 signals attenuate TCR-mediated transcriptomic changes
Source: iScience. 2024 Jun 12;27(7):110253. doi: 10.1016/j.isci.2024.110253 (PMC11253514; doi:10.1016/j.isci.2024.110253)
Supplement: Document S1. Figures S1–S4 [file mmc1.pdf]

iScience, Volume *27*

Supplemental information

BTLA and PD-1 signals attenuate

TCR-mediated transcriptomic changes

Muhammad Zainul Arifin, Judith Leitner, Donagh Egan, Petra Waidhofer-Söllner, Walter Kolch, Vadim Zhernovkov, and Peter Steinberger

Supplemental Information

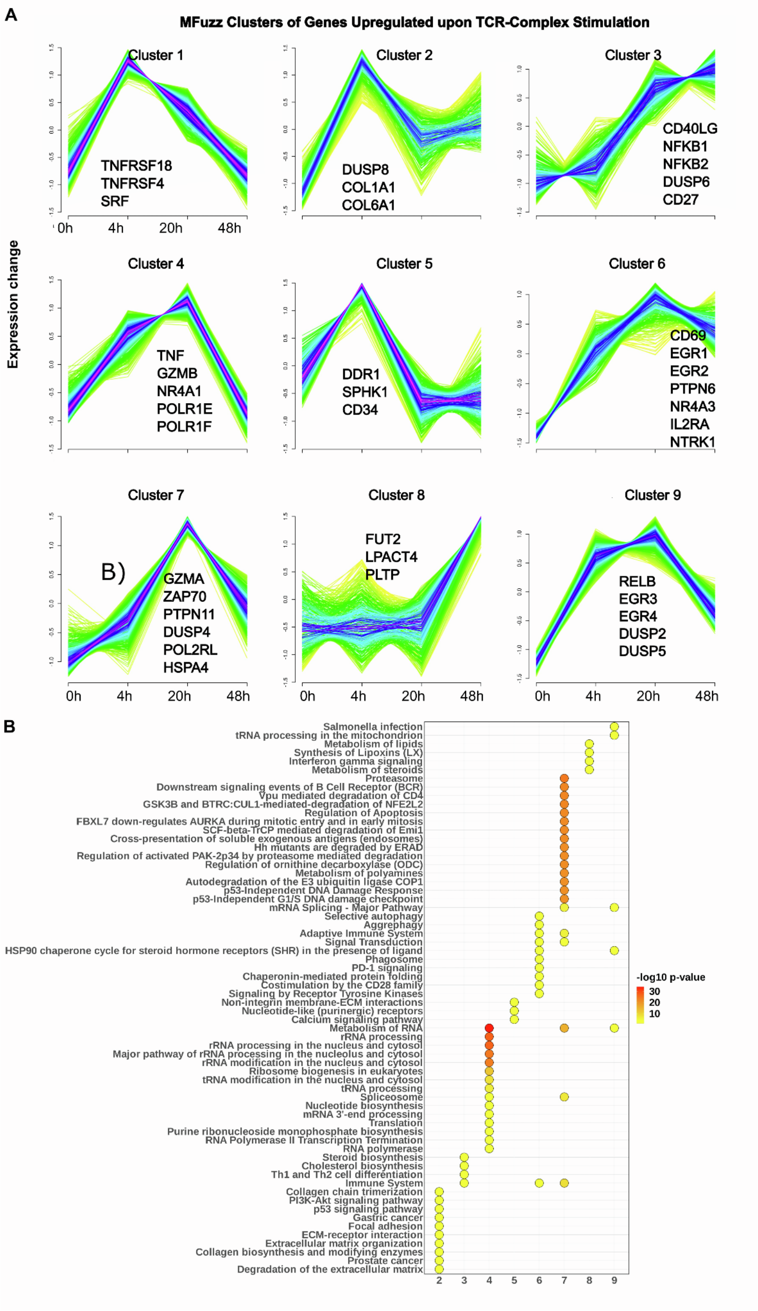

Figure S1. MFuzz Clustering revealed different clusters exhibiting distinct signalling pathways upon TCR-complex stimulation, related to Figure 2.

A.) Clustering of genes upregulated upon TCR-complex stimulation based on gene expression temporal pattern with MFuzz.

B.) Bubble plot of over-representation analysis results of MFuzz clusters with KEGG and REACTOME databases. The 15 most statistically significant pathways for each cluster were gathered, with a significance threshold of p-value < 0.05. Subsequently, each collected pathway was examined across all clusters, and significant pathways were plotted.

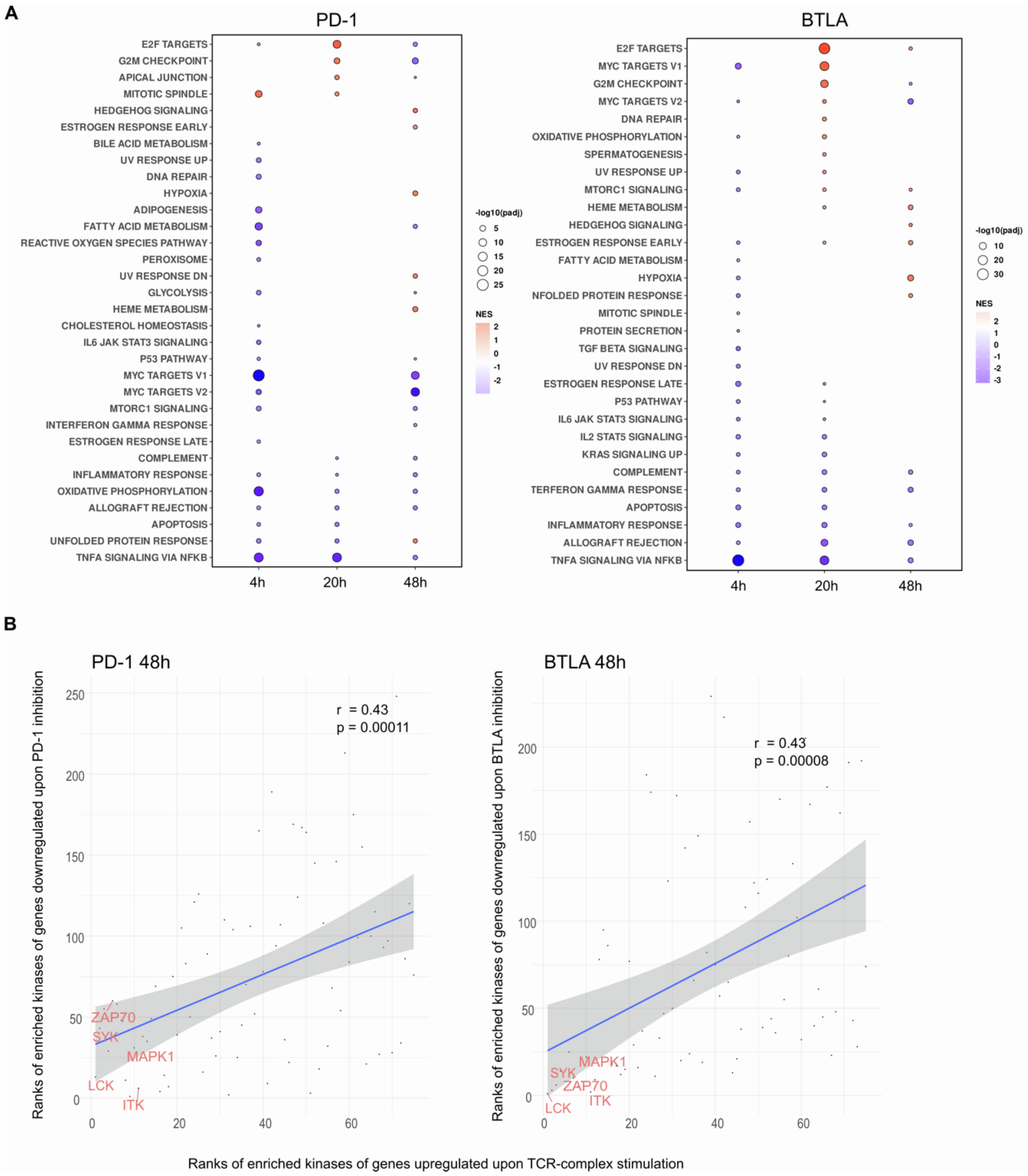

Figure S2: Pathways induced during T cell activation were suppressed by PD-1 and BTLA signals, related to Figure 4.

A.) Bubble plot of gene set enrichment analysis results using the Hallmark database used to identify differentially enriched pathways (adjusted p-value < 0.05) for PD-1 and BTLA for all time points.

B.) Correlation analysis between kinases rank from kinase enrichment analysis on genes significantly upregulated upon TCR-complex stimulation and genes significantly downregulated by PD-1/BTLA inhibition. The top 75 highest-ranked kinases from the kinase enrichment analysis results of genes upregulated upon TCR-complex stimulation were used for the 48-hour time point.

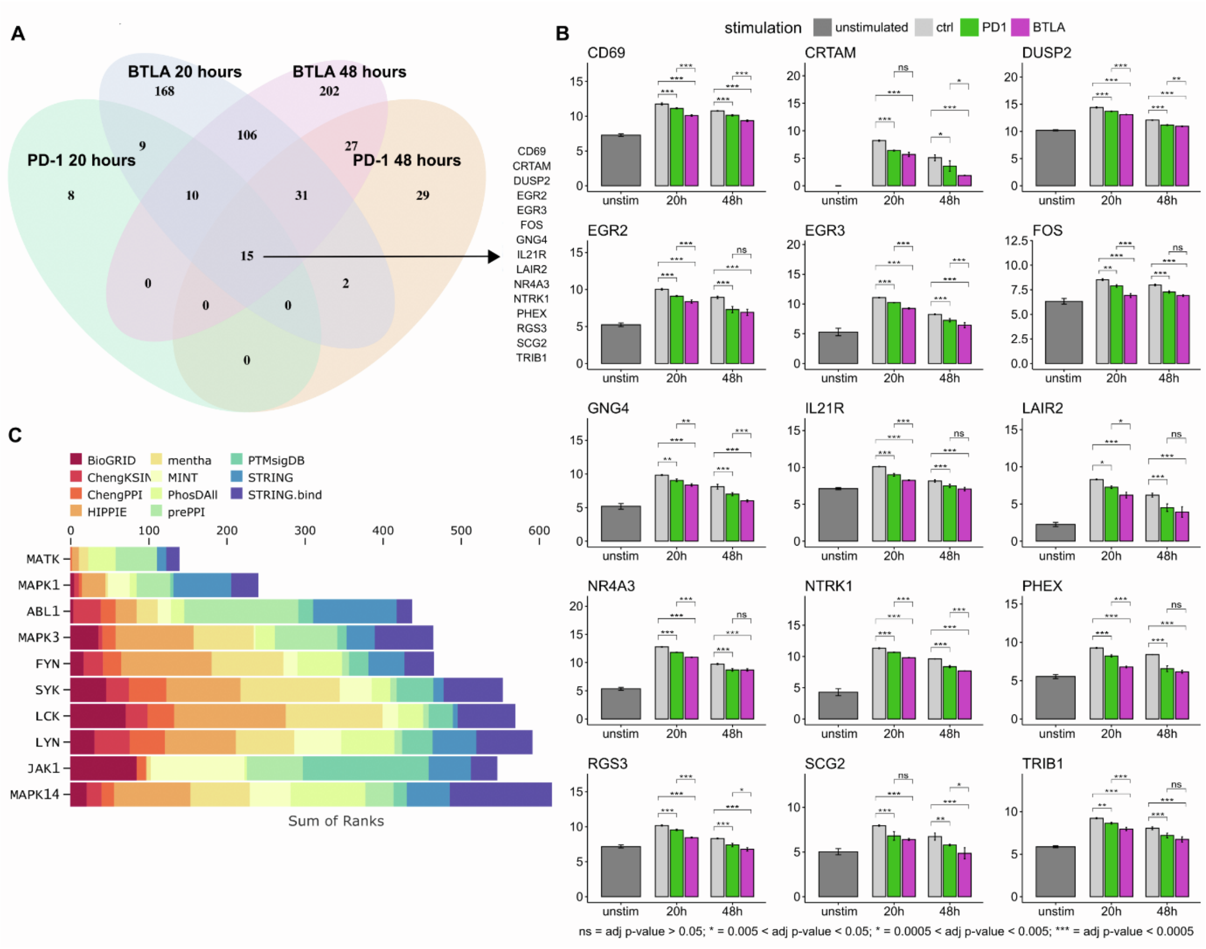

Figure S3: 15 genes were significantly downregulated upon engagement of PD-1 and BTLA at the 20 and 48-hour time points, related to Figure 4.

A.) Downregulated genes upon PD-1 and BTLA inhibition at 20 and 48 hours.

B.) Normalized log2 expression of downregulated genes upon PD-1 (green) and BTLA (magenta) inhibition at 20 and 48 hours with differential expression statistics. Ns adj p> 0.05; * = 0.005 < adj p-value < 0.05; * = 0.0005 < adj p-value < 0.005; *** = adj p-value < 0.0005

C.) Kinase enrichment analysis of common differentially expressed genes in PD-1 and BTLA stimulated samples at 20 and 48 hours. The ten kinases with the highest aggregated rank from 11 kinase databases were plotted.

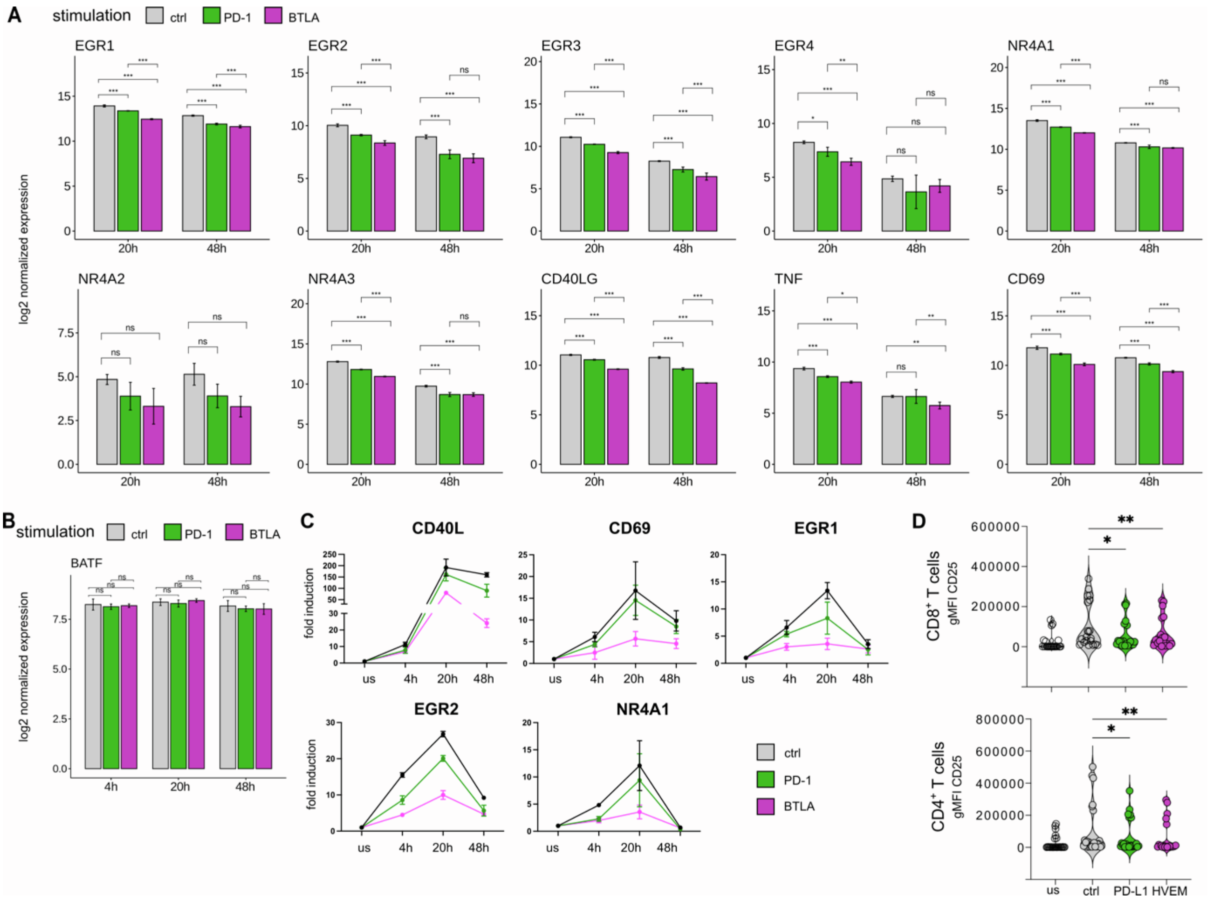

Figure S4: **PD-1 and BTLA signals strongly inhibited activation-induced genes but also several transcription factors, related to Figure 4.**

A.) Normalized log2 expression of EGR1, EGR2, EGR3, EGR4, Nr4a1, Nr4a2, Nr4a3, CD40LG, TNF, and CD69 at 20 and 48 hours with differential expression statistics.

B.) Normalized log2 expression of BATF at 20 and 48 hours with differential expression statistics. Ns, p > 0.05; * = 0.005 <p-value < 0.05; * = 0.0005 < p-value < 0.005; *** = p-value < 0.0005

C.) Real-Time PCR data of selected genes at 4h, 20h and 48h of stimulation. Data was normalized to the unstimulated condition and is depicted as fold induction.

D.) Human PBMCs were stimulated for 72h in presence of TCS-control, TCS-PDL1 and TCS-HVEM or left unstimulated (us).CD25 expression was determined in gated CD4 and CD8 T cell subset (gMFI; geometric mean fluorescence intensity). Data of 7 donors (n=7) each performed in duplicates or triplicates is shown. One-way Anova followed by Dunnett's multiple comparison (compared to control stimulation) was used. *p ≤ 0.05; **p ≤ 0.01.
